# Temporal Dynamics of PD-L1 Surface in RKO Cells: Characterizing the Reversible Impact of the Small-Molecule Dimerizer BMS-202

**DOI:** 10.3390/biomedicines14081659

**Published:** 2026-07-23

**Authors:** Gohar Sevoyan, Daniel Polianczyk, Siranuysh Grabska, Hovakim Grabski, Ruben Abagyan, Zaruhi Karabekian

**Affiliations:** 1 L.A. Orbeli Institute of Physiology, National Academy of Sciences, Yerevan 0028, Armeniazkarabekian@yahoo.com (Z.K.); 2 Skaggs School of Pharmacy and Pharmaceutical Sciences, University of California, La Jolla, San Diego, CA 92093-0657, USA

**Keywords:** immune checkpoint inhibitors, cancer, immune system, assay

## Abstract

**Background/Objectives.** Programmed death-ligand 1 (PD-L1) is a critical immune checkpoint protein that enables tumors to evade immune surveillance by suppressing T cell activation. Monoclonal antibodies are currently used to modulate PD-1/PD-L1 interactions. However, several immune-associated adverse effects were ascribed to those treatments. This led to the necessity for small-molecule alternatives like BMS-202. **Methods.** In this study, we investigated the temporal dynamics of cell-surface PD-L1 in response to the small-molecule dimerizer BMS-202. Treatment with a non-cytotoxic concentration of BMS-202 at 5 µM triggered a transient reduction in surface PD-L1 concentration, reaching its lowest level at 15 min. In the attempt to characterize the fate of PD-L1 following exposure to the BMS-202 dimerizer, we employed a low-pH wash internalization assay. **Results.** The results demonstrated a transient increase in intracellular PD-L1 within 5–15 min of compound exposure, followed by rapid recovery of surface PD-L1 levels. These findings suggest that BMS-202-induced changes in surface PD-L1 are acute and reversible, with levels returning to baseline within 24 h post-exposure. **Conclusions.** These findings reveal a dynamic regulatory mechanism where small-molecule-induced dimerization triggers rapid protein trafficking and transient surface depletion. Understanding these temporal dynamics is essential for the development of next-generation small-molecule immune checkpoint inhibitors.

## 1. Introduction

Programmed death-ligand 1 (PD-L1) is a critical immune checkpoint protein that plays a pivotal role in cancer biology by mediating immune evasion and promoting tumor progression. PD-L1 binds to its receptor Programmed death-1 (PD-1) on T cells and other immune cells, transmitting inhibitory signals that suppress T cell proliferation and effector functions, thereby enabling tumors to escape immune surveillance and establish immune tolerance [1,2]. This immune checkpoint pathway has become a major focus in cancer immunotherapy, with PD-1/PD-L1 blockade therapies revolutionizing treatment paradigms and achieving durable anti-tumor responses in various malignancies [1,3].

Beyond its well-characterized role in immune suppression, PD-L1 also exhibits tumor-intrinsic functions that contribute to cancer cell proliferation, invasion, metastasis, and resistance to chemotherapy. Studies have demonstrated that PD-L1 expression correlates with epithelial-mesenchymal transition (EMT) and enhances tumor stemness through signaling pathways such as mTORC1, thereby promoting tumor aggressiveness [1]. Moreover, PD-L1 can modulate cancer cell survival independently of the immune system by regulating responses to DNA damage and type I interferon signaling, further enhancing tumor resistance to therapies [4].

In addition to the membrane-bound form, soluble and exosomal forms of PD-L1 have been detected in the circulation of cancer patients. These variants add complexity to PD-L1 biology by contributing to immunosuppression, serving as biomarkers for tumor progression, and predicting responses to immunotherapy [2,5]. Understanding the diverse molecular mechanisms and clinical implications of PD-L1-including its soluble and exosomal forms is essential for optimizing therapeutic strategies and improving patient outcomes.

A promising class of small molecules, exemplified by BMS-202, functions through a unique mechanism: they induce the dimerization of PD-L1 at the PD-1 binding interface [6]. This dimerization not only blocks the interaction with PD-1 but is also hypothesized to trigger the internalization and subsequent degradation or recycling of the protein. However, the temporal dynamics of this process, specifically how quickly surface PD-L1 is depleted and how rapidly the cell restores its surface expression, remain insufficiently characterized.

BMS-202 [7] is a PD-L1 inhibitor that induces dimerization through the PD-1 binding interface, representing a mechanism beyond simple competitive binding. This mode of action was elucidated by NMR and confirmed by X-ray crystallography, which revealed key interactions stabilizing the inhibitor at the PD-L1 interface [8].

Unlike the widely adopted monoclonal antibody (mAb) therapies, BMS-202 exerts its effect by directly binding to PD-L1, thereby blocking its interaction with PD-1 and effectively disarming a key mechanism by which cancer cells evade immune surveillance [9]. Preclinical studies have demonstrated its potent in vitro activity, inhibiting the proliferation of PD-L1-positive cancer cells and, crucially, restoring T cell function by rescuing interferon-gamma (IFN-γ) production [10].

The development of small-molecule inhibitors like BMS-202 is particularly noteworthy as it addresses several limitations inherent to antibody-based treatments, offering potential advantages such as oral bioavailability, enhanced tumor penetration, reduced production costs, and potentially lower immunogenicity. Furthermore, in vivo models have shown that BMS-202 elicits robust antitumor activity through comprehensive immunomodulation, characterized by an increase in cytotoxic T cell populations and a reduction in immunosuppressive regulatory T cells within the tumor microenvironment [11].

In this study, we utilize BMS-202 as a reference compound to investigate the dynamic regulation of cell-surface PD-L1. By developing a refined flow cytometric internalization assay featuring a low-pH wash (acid wash) step, we aim to map the kinetics of PD-L1 trafficking from the plasma membrane to intracellular compartments. Our objective is to characterize the transient depletion and rapid recovery of PD-L1 expression, providing essential mechanistic insights into the cellular fate of small-molecule-induced dimers, a critical step for optimizing the dosing and efficacy of next-generation immune checkpoint therapies.

## 2. Materials and Methods

### 2.1. Cells

Several cancer cell lines were used in this study. These cell lines were selected based on data from the Human Protein Atlas due to their documented expression of PD-L1. Non-cancer lines were used as controls. For in vitro testing, RKO (colon cancer cells, high expressors of PD-L1), MDA-MB-231 (triple-negative breast cancer cells, moderate expressors of PD-L1), or HaCaT cells (normal human keratinocytes, very low PD-L1 expressors) were used. The cell lines were procured from the American Type Culture Collection (ATCC, Manassas, VA, USA). All cells were cultured in Dulbecco’s Modified Eagle Medium (DMEM) supplemented with 5% (*v*/*v*) fetal bovine serum (Gibco, Thermo Fisher Scientific, USA), 100 U/mL penicillin, and 100 μg/mL streptomycin. Cultures were maintained at 37 °C in a humidified atmosphere containing 5% CO_2_.

*Cytotoxicity assessments*

MTT assay

The MTT assay (3-(4,5-dimethylthiazol-2-yl)-2,5-diphenyltetrazolium bromide) was performed to determine the highest non-cytotoxic concentration of BMS-202. The assay is based on the conversion of MTT into insoluble purple formazan crystals by metabolically active cells [12]. Cells were seeded into 96-well plates at a density of 1 × 10^4^ cells per well and allowed to adhere for 24 h. The medium was then replaced with a fresh complete medium containing BMS-202 at final concentrations of 0.1, 0.3, 1, 3, 10, 30, and 100 μM. Cells cultured in complete medium containing 0.5% (*v*/*v*) DMSO alone served as the vehicle control. The final concentration of DMSO was maintained at 0.5% (*v*/*v*) in all experimental and control wells. All groups were incubated for 48 h. The treatment medium was replaced with fresh complete medium, and 20 μL of MTT solution (5 mg/mL in PBS) was added to each well. Following incubation for 3 h at 37 °C, the medium was aspirated, the formazan crystals were solubilized for 20 min in 100 μL of 100% DMSO, and absorbance was measured at 570 nm using a SYNERGY H1 microplate reader (Agilent Technologies, Santa Clara, CA, USA ). MTT assay data were obtained from three independent experiments, each performed in technical triplicate. Results are presented as mean ± standard deviation (SD). Statistical analysis was performed using one-way analysis of variance (ANOVA) to evaluate differences among BMS-202 concentrations. A *p*-value < 0.05 was considered statistically significant.

Annexin V–FITC/Propidium Iodide (PI) double-staining assay

The cytotoxicity of BMS-202 was confirmed by orthogonal methods, specifically an Annexin V/Propidium Iodide (PI) apoptosis assay. RKO cells were seeded into 12-well plates and allowed to attach overnight in DMEM supplemented with 5% FBS (antibiotic-free). When cultures reached approximately 70% confluency, cells were treated with BMS-202 (0.1, 0.3, 1, 3, 10, 30, and 100 μM). Vehicle-treated cells served as negative controls, while positive control cells were incubated with 2.5% or 5% DMSO. A total of 1 × 10^5^ cells/per treatment were collected by centrifugation. Pellets were washed once with cold PBS. Cells were resuspended in 1× Binding Buffer, 100 µL per sample. A pre-prepared Annexin V–FITC/PI staining mix was added to each sample, 50 µL, followed by gentle mixing. Samples were incubated for 20 min at room temperature in the dark. Samples were washed and analyzed within 1 h using a flow cytometer.

### 2.2. Flow Cytometric Assessments

The levels of PD-L1 on the cell surface following treatment with the test reagents were assessed by flow cytometry [13]. Based on the MTT assay results, a concentration of 5 μM was selected as the maximum non-cytotoxic dose. For this and the following experiments, we used only RKO cells as a high expressor PD-L1 cell line. Cells were treated with this concentration for 5, 15, 30, and 60 min, as well as 24 h. At each time point, cells were harvested, washed with PBS, and incubated with an anti-PD-L1 primary antibody (14-5983-82, Invitrogen, Carlsbad, CA, USA) for 60 min at 4 °C. Subsequently, cells were stained with a goat anti-mouse Alexa Fluor 488-conjugated secondary antibody (ab150113, Abcam, Cambridge, UK) for 30 min at 4 °C. After two additional PBS washes, samples were analyzed using a CyFlow^®^ Cube 6 flow cytometer (Goerlitz, Germany), acquiring at least 10,000 events per sample [14]. Data presented as mean value ± SE of three independent experiments.

Data analysis was conducted using FlowJo software (version 11) (FlowJoTM Software for Windows [Software Application] Version 11. Ashland, OR: Becton, Dickinson and Company; 2023., n.d.).

### 2.3. PD-L1 Internalization Assay

**Figure 1:**
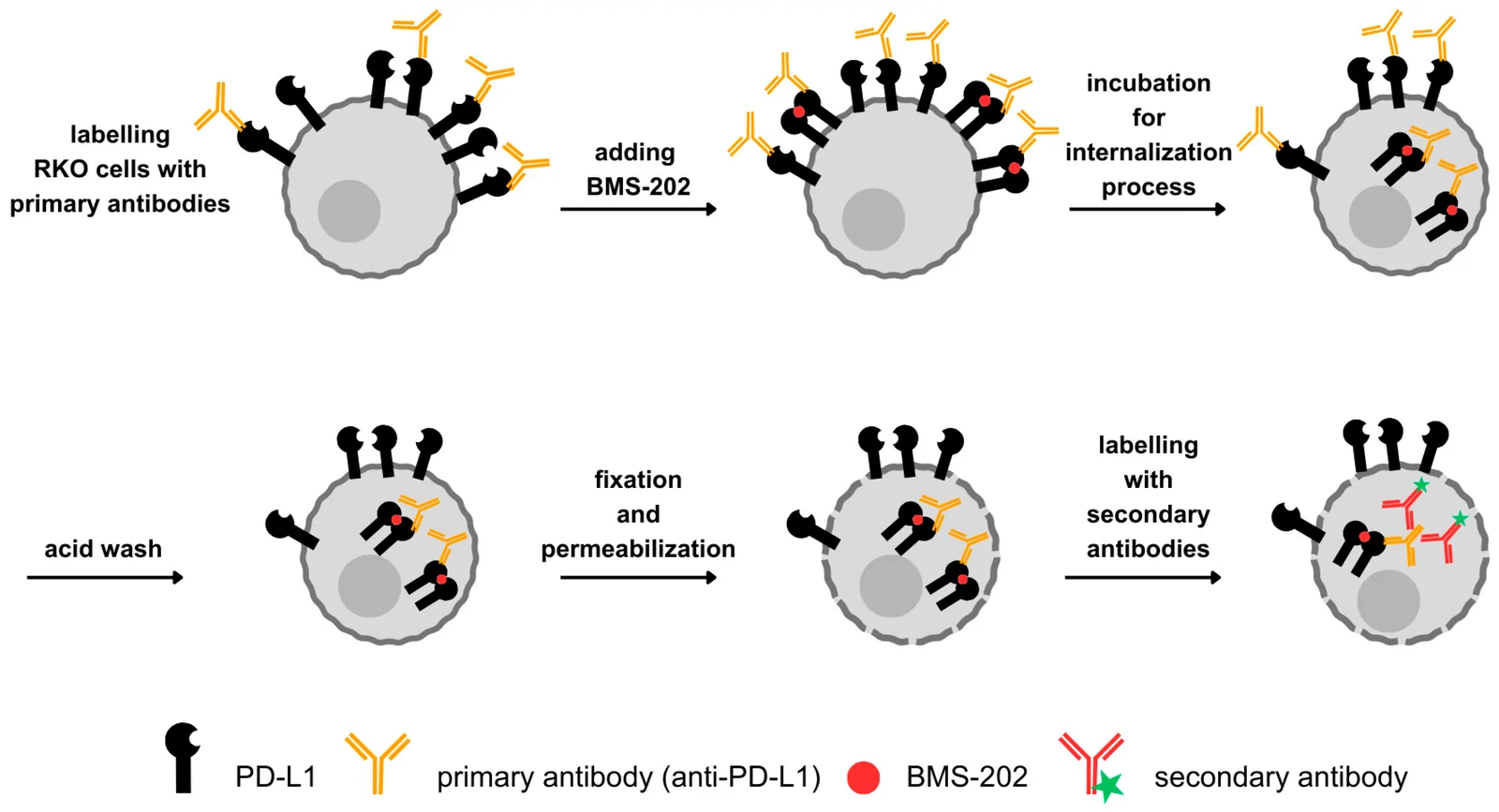
Schematics of PD-L1 internalization assay.

Cells were collected, washed, and transferred to 1.5 mL tubes. Cell surface PD-L1 was labeled with unconjugated anti-PD-L1 (14-5983-82, clone MIH1, Invitrogen; or MAB1561, clone 130021 [15], R&D Systems: or ab205921, clone 28-8, Abcam) for 30 min at 37 °C and washed twice to remove unbound antibodies. Washed cells were resuspended in DMEM and incubated with the tested compound at 37 °C. Samples were removed at the indicated time points and placed in pre-cold (4 °C) centrifuge to stop internalization. Cells were washed twice with a cold low pH buffer (Glycine-HCl Buffer, 0.1 M, pH 3.0) for 2 min at 4 °C, aspirated, and then washed with cold PBS. To assess the viability after acid wash, Trypan Blue was used to exclude membrane disruption. Viability drops by 10 percentage points (from 95% to 85%). After cells were fixed with 2% PFA for 10 min.; cells were then permeabilized with 0.2% Tween 20 for 10 min at RT. Alexa Fluor-488-conjugated secondary antibodies (ab150113 or ab150077, Abcam) were added for 30 min at 4 °C to visualize the internalized anti-PD-L1 antibody. Lastly, stained cells were washed with PBS and analyzed by flow cytometry (Figure 1).

**Figure A1:**
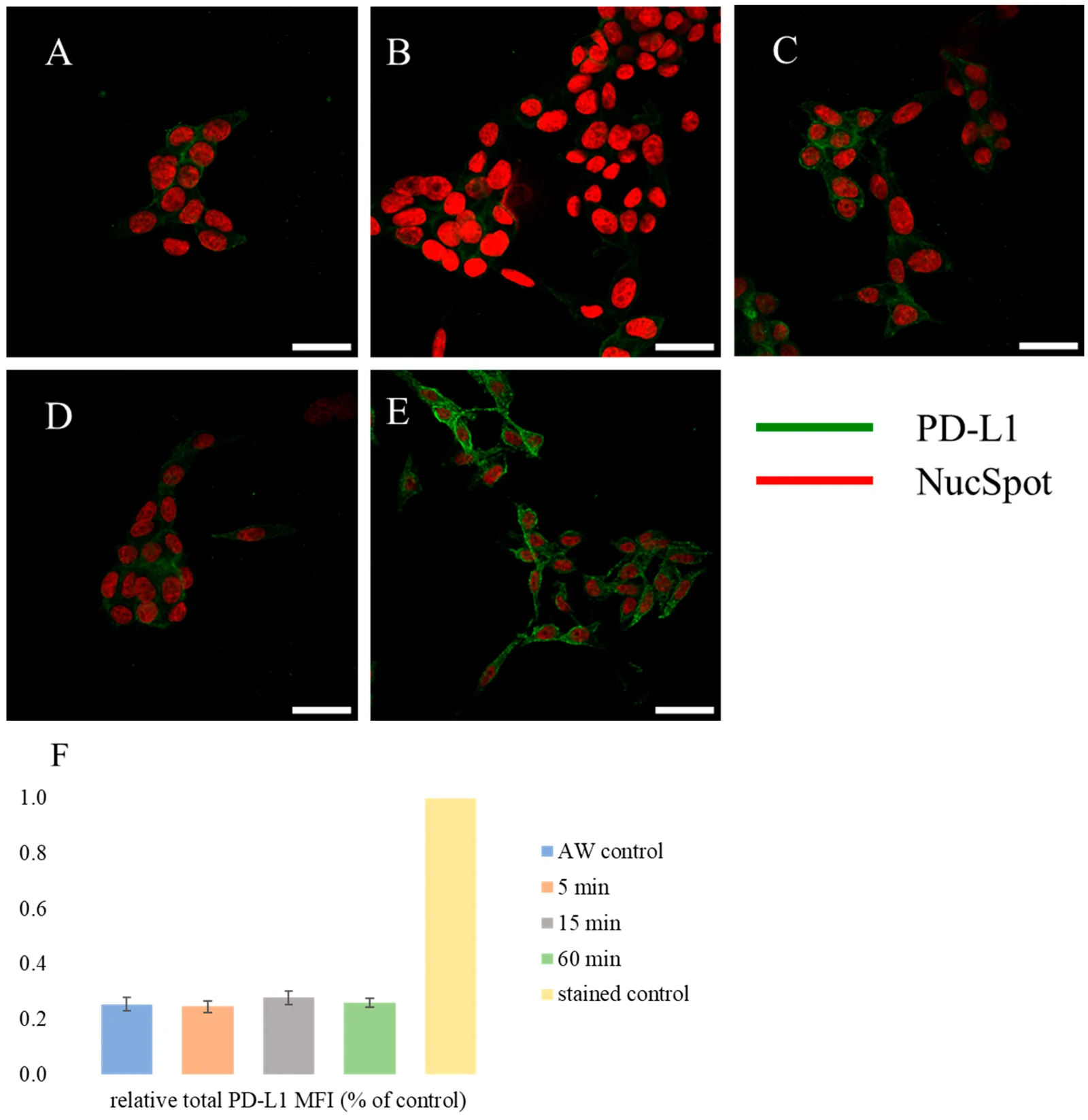
RKO cells confocal images in acid wash assay. (**A**) acid wash-stained control, (**B**) 5 min treated, (**C**) 15 min treated, (**D**) 60 min treated, (**E**) stained control. Scale bar—30 µm. (**F**) representation of relative MFI for total (surface and intercellulat) PD-L1. Nine individual fields were used to calculate fluorescence signals ± SD.

Additionally, we used confocal microscopy to assess the fluorescence signal for total PD-L1 (surface and internalized) (Figure A1). RKO cells were seeded onto 13 mm glass coverslips coated with gelatin placed in 12-well Corning plates. Cell surface PD-L1 was labeled with unconjugated anti-PD-L1 (14-5983-82, Invitrogen) for 30 min at 37 °C and washed twice to remove unbound antibodies. Washed cells were resuspended in DMEM and incubated with the tested compound at 37 °C. Samples were removed at the indicated time points and placed on ice to stop internalization. Cells were washed twice with a cold low pH buffer (Glycine-HCl Buffer, 0.1 M, pH 3.0) for 2 min, aspirated, and then washed with cold PBS. After cells were fixed with 2% PFA for 10 min. Cells were then permeabilized with 0.2% Tween 20 for 10 min at RT. Alexa Fluor-488-conjugated secondary antibodies (ab150113, Abcam) were added for 60 min at RT. Bovine serum albumin (2% BSA) was used for blocking. The coverslips with stained cells were mounted on the glass microscopic slides with a drop of Mowiol.

The samples were imaged with confocal laser scanning microscope Leica SPE (Leica Microsystems Inc., Wetzlar, Germany) equipped with a solid-state laser for excitation and PMT detectors. Images within each sample set were captured using identical instrument settings. The acquisition was performed using several sequences to minimize artifacts. The images were analyzed with open source software FIJI [16].

## 3. Results

BMS-202 occupies a distinct profile within the immune checkpoint blockade landscape, establishing itself as a significant compound in this evolving field. However, BMS-202 is subject to certain limitations:

1. Variable Potency: While BMS-202 shows nanomolar potency in direct protein–protein interaction assays (e.g., 18 nM), its effectiveness in more complex cellular assays, such as inhibiting cell proliferation, often falls into the micromolar range (e.g., 10–15 μM) (BMS-202|PD-1/PD-L1 Inhibitor|MedChemExpress, n.d.).
2. Lack of Clinical Approval: Despite promising preclinical data, no small-molecule PD-L1 inhibitors, including BMS-202, have yet received FDA approval for cancer treatment.

### 3.1. Assessment of Cytotoxicity of Experimental Compounds

**Figure 2:**
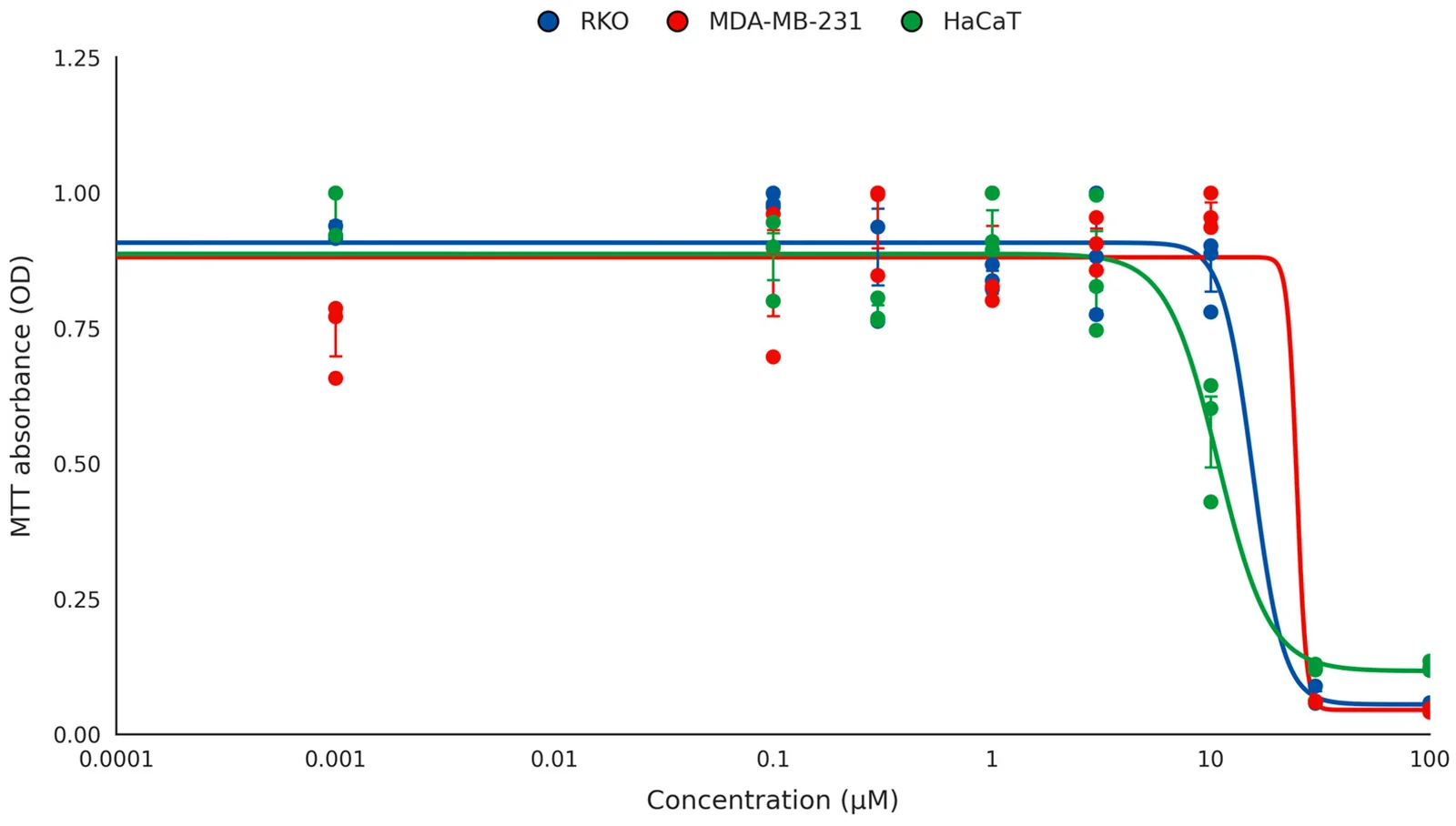
Effect of BMS-202 on metabolic activity. Metabolic activity of RKO, MDA-MB-231, and HaCaT cells was assessed by the MTT assay following treatment with increasing concentrations of BMS-202. Data (n = 3) are presented as mean ± SD of absorbance (OD). A dose-dependent decrease in metabolic activity was observed in all tested cell lines.

To determine the maximum non-cytotoxic concentrations of BMS-202 for subsequent functional assays, an MTT assay was conducted. This colorimetric assay evaluates cell metabolic activity by measuring mitochondrial function, specifically through the reduction in MTT by dehydrogenase enzymes to form insoluble purple formazan crystals [12]. Since mitochondrial activity is an inherent property of live cells, it is often used to evaluate cell viability. The MTT assay results demonstrated that BMS-202 did not significantly affect the metabolic activity of MDA-MB-231 or HaCaT cells at concentrations below 10 μM, with cell viability remaining comparable to untreated controls. At higher concentrations (≥30 μM), BMS-202 significantly reduced cell viability in a concentration-dependent manner. One-way ANOVA demonstrated statistically significant differences among the tested concentrations in all three cell lines (*p* < 0.01). The strongest cytotoxic effect was observed at 30 and 100 μM, where cell viability was markedly reduced compared with lower concentrations (Figure 2).

### 3.2. Assessment of the Fate of the Cell Lines Following Treatment with BMS-202

**Figure 3:**
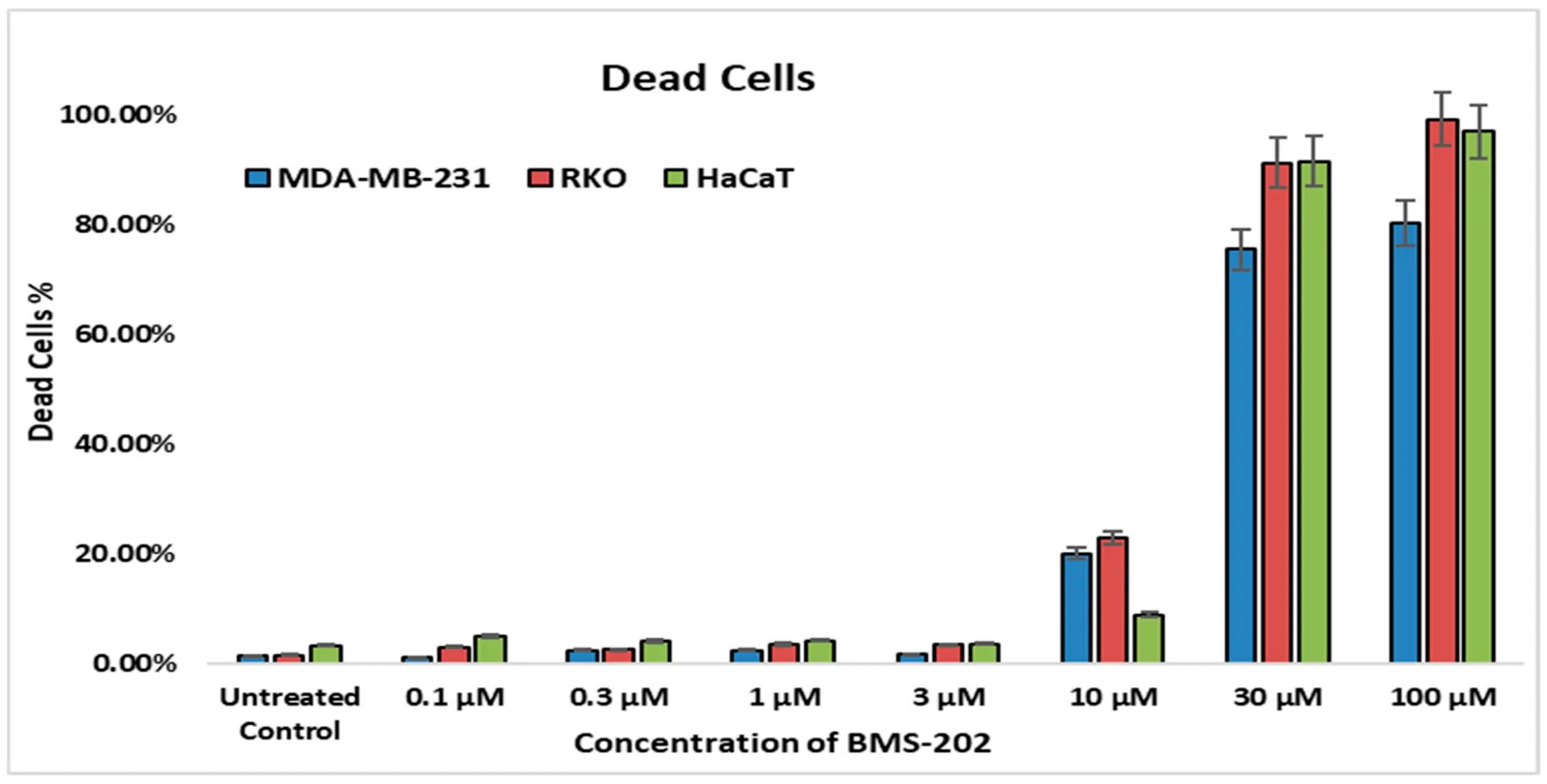
BMS-202 induces dose-dependent cell death in cancer and non-malignant cells. Quantification of dead cells, defined as the sum of late apoptotic (Q2) and necrotic (Q1) populations. Data are presented as mean ± SD from three independent experiments (n = 3).

**Figure A2:**
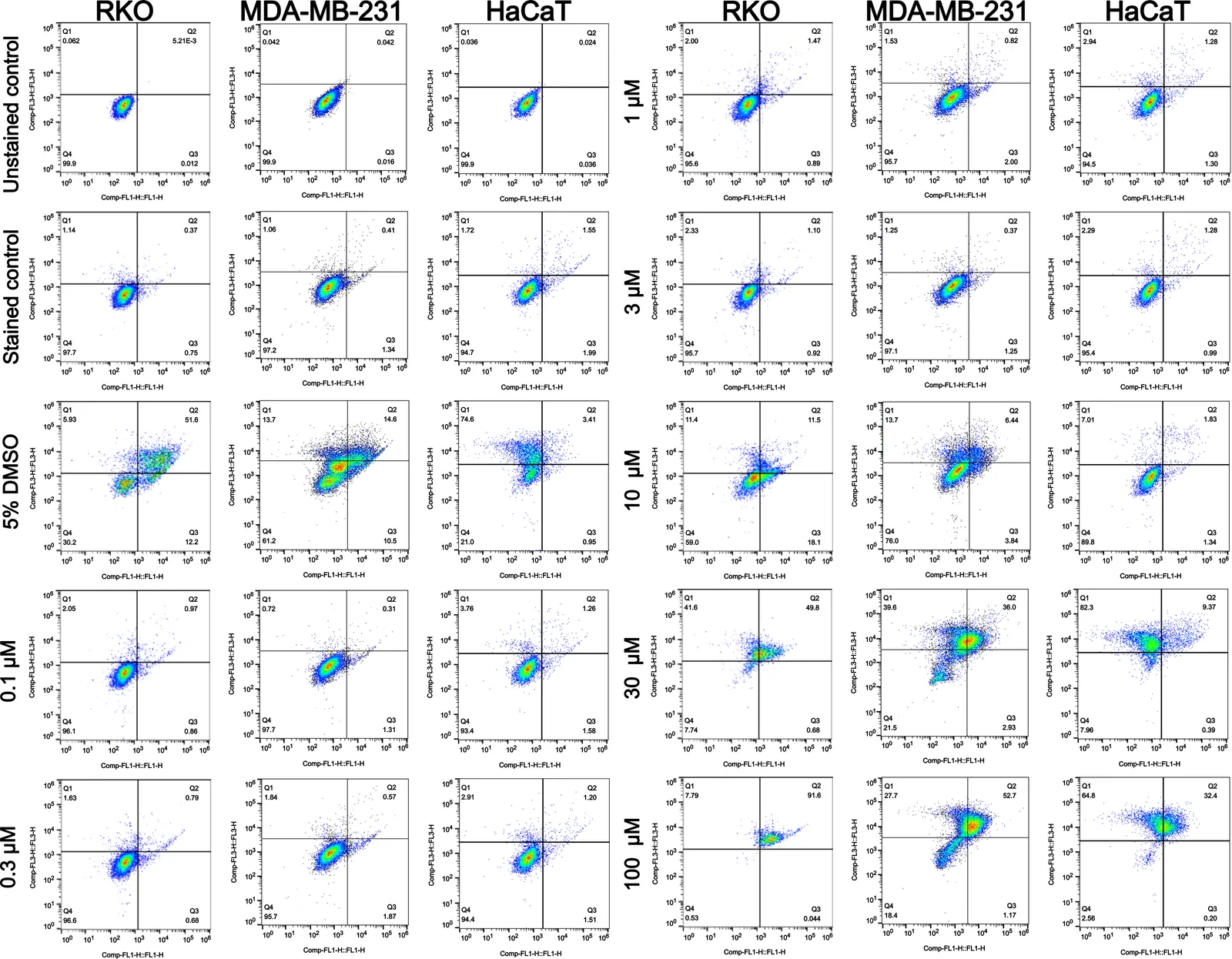
BMS-202 induces dose-dependent cell death in cancer and non-malignant cells. Representative flow cytometry dot plots of Annexin V/PI staining in MDA-MB-231, RKO and HaCaT cells treated with the indicated concentrations of BMS-202. Quadrant distribution identifies live (Annexin V^−^/PI^−^; Q4), early apoptotic (Annexin V^+^/PI^−^; Q3), late apoptotic (Annexin V^+^/PI^+^; Q2), and necrotic (Annexin V^−^/PI^+^; Q1) populations.

To understand the processes taking place upon exposure of the cells to BMS-202, induction of apoptosis was evaluated by Annexin V/Propidium Iodide (PI) flow cytometry in RKO and MDA-MB-231 colorectal and breast cancer cells, alongside non-malignant HaCaT keratinocytes. In untreated and stained control samples, >97% of cells were Annexin V^−^/PI^−^, indicating low basal apoptosis (Figure A2 and Figure 3).

BMS-202 treatment resulted in a concentration-dependent increase in cell death across all three cell lines. At low concentrations (0.1–3 μM), viability remained largely unaffected (>95%), with only marginal increases in early apoptotic fractions and minimal late apoptosis or necrosis.

At 10 μM, a measurable reduction in viability was observed in cancer cells (to ~76–80%), accompanied by increased late apoptotic and necrotic populations. In contrast, HaCaT cells exhibited a comparatively modest response at this concentration, maintaining >90% viability.

A pronounced cytotoxic effect was evident at 30 μM, where viability in RKO and MDA-MB-231 cells declined to approximately 20–25%, with a substantial accumulation of late apoptotic and necrotic cells. Notably, HaCaT cells also demonstrated marked sensitivity at this concentration, with viability decreasing to below 10%.

At 100 μM, extensive apoptosis was observed in all cell lines, characterized by a dominant late apoptotic population and minimal residual viability. Collectively, these findings indicate that BMS-202 induces robust, dose-dependent cell death, with limited effects at low micromolar concentrations but pronounced cytotoxicity at ≥30 μM in both malignant and non-malignant cells. Distinct cytotoxic profiles of BMS-202 are consistent with the results obtained from MTT viability assays.

### 3.3. Flow Cytometric Assessment of PD-L1 Expression on the Surface of PD-L1-Expressing Cell Lines Following Treatment with BMS-202

**Figure 4:**
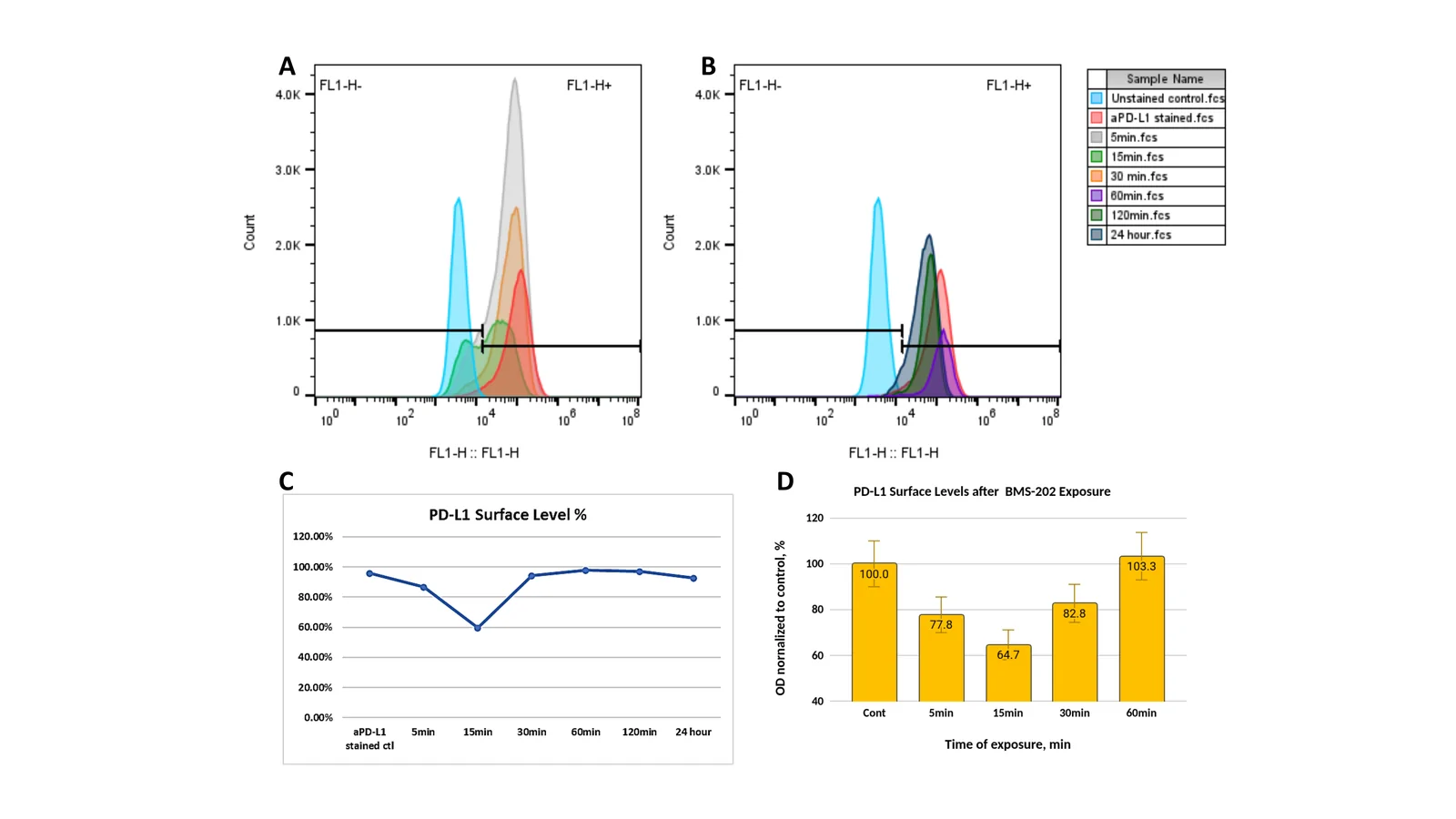
Temporary changes in RKO cells’ surface PD-L1 levels were observed upon exposure to BMS-202. RKO cells were treated with 5 μM BMS-202 for 5, 15, 30, 60 min and 24 h. Cells were stained for PD-L1 and analyzed by flow cytometry. (**A**) Representative flow cytometry histograms for early time points; (**B**) representative flow cytometry histograms for late time points; (**C**) and corresponding mean fluorescence intensity (MFI, FL1-H) for the indicated time points; (**D**) PD-L1 surface expression (n = 3) was quantitated as OD normalized to the untreated controls (mean ± SE).

Preliminary testing of PD-L1 surface level testing of these three cell lines supported a known expression level. However, the modulation of the detected levels of PD-L1 on MDA-MB-231 was less pronounced. Therefore, we continued our studies using solely RKO cell lines. To determine the impact of BMS-202 on PD-L1 surface expression in RKO cells, we conducted flow cytometric analysis following BMS-202 treatment. All cells were exposed to a concentration of 5 μM, previously identified via MTT assay as the highest non-cytotoxic dose. RKO cells were treated for 5, 15, 30, and 60 min, as well as a 24 h recovery time point. At each time point, both treated and untreated control cells were stained under identical conditions using an anti-PD-L1 antibody. Flow cytometric acquisition captured at least 10,000 events per sample. Results are presented as histograms (Figure 4A,B) and bar graphs with corresponding mean fluorescence intensity (MFI) values (Figure 4C,D).

Treatment with BMS-202 triggered a transient reduction in PD-L1 surface levels, with the lowest amount determined by the mean fluorescence intensity (MFI) observed at 15 min. This was followed by a gradual increase at 30 and 60 min, ultimately reaching the initial levels of the control samples at the final time point (24 h of incubation). Interestingly, the percent of PD-L1 surface-positive cells remains relatively steady after 30–60 min of exposure (Figure 4D), whereas MFI demonstrates a tendency to decrease within the same time frame (Figure 4C). This pattern suggests a novel BMS-202 specific modulation of PD-L1 dynamics, rendering statistical proof.

### 3.4. Internalization of PD-L1

Upon exposure of RKO cells to the test compounds, we detected a temporal decrease in PD-L1 molecules on the surface of the cells, followed by recovery to the initial levels. Such a decrease can be induced by the dimerization of individual PD-L1 molecules. Studies showed that dimerization of PD-L1 molecules induced by any agent leads to the generation of a non-functional molecule, which is being internalized [8]. Internalized dimers can undergo recycling and return to the surface of the cells [17], or degradation [18].

BMS-202 is a known dimerization agent for PD-L1 molecules [9]. To prove internalization of dimerized molecules, we conducted an internalization assay detailed in Materials and Methods.

**Figure 5:**
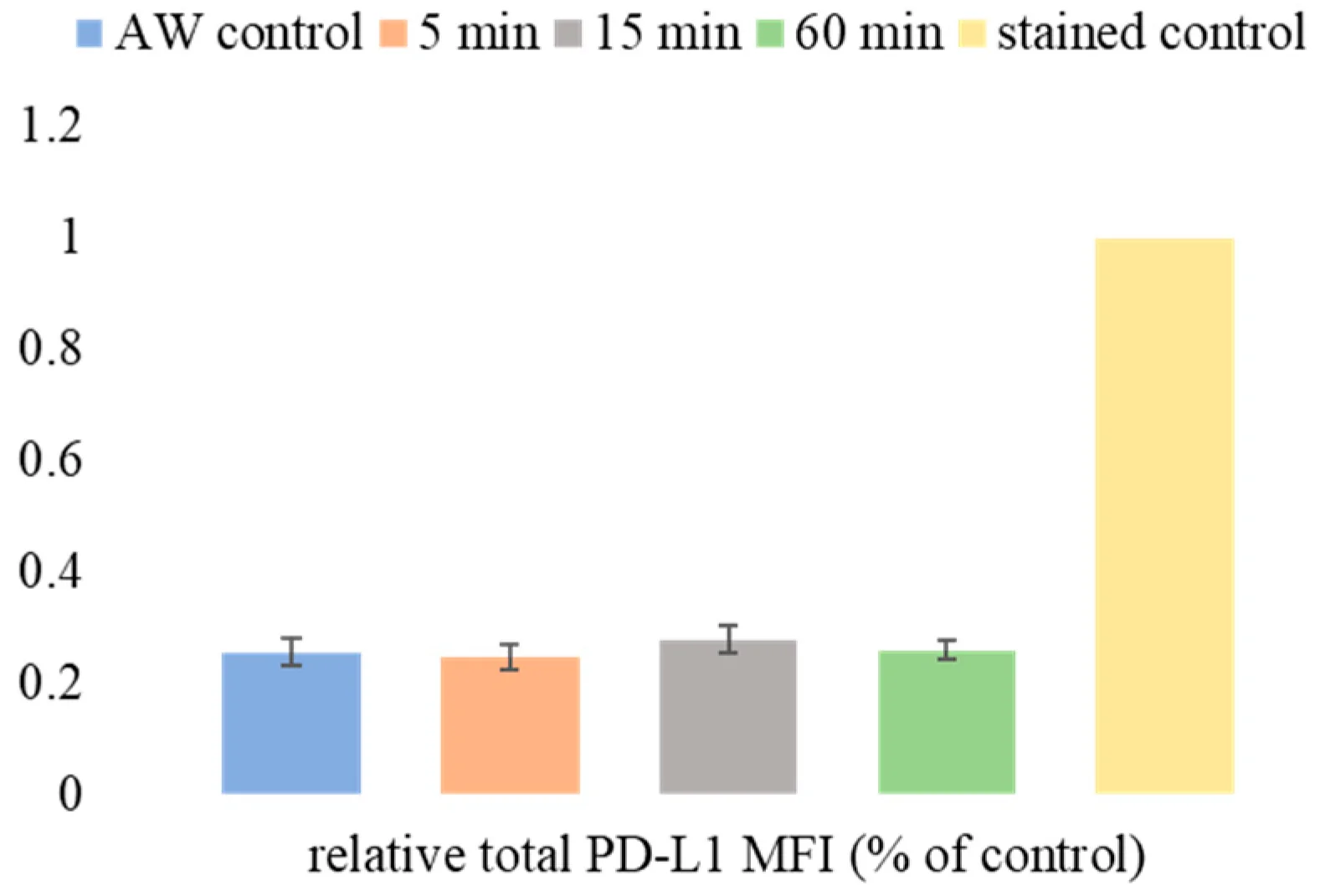
Assessment of total PD-L1 signal after acid-wash procedure. RKO cells were incubated with BMS-202 for the indicated periods and subjected to acid wash (see method details in Figure 1). RKO cells were stained with unconjugated PD-L1 abs and treated with 5 μM of BMS-202 for 5, 15, and 60 min. After fixation/permeabilization, cells were stained with secondary antibodies and analyzed by flow cytometry. Results are represented as mean of MFI value ± SD (n = 3). Data was analyzed by ANOVA, followed by the Bonferroni post hoc test.

Acid-wash controls reproducibly demonstrated the removal of approximately 80% of the anti-PD-L1 antibody. Cells exposed to BMS-202 for 5 min showed little change in PD-L1 signal, suggesting that this time point is too early to detect the internalization of labeled, dimerized PD-L1 molecules. After 15 min of BMS-202 treatment, a modest increase in intracellular PD-L1 signal was observed, although the difference was not statistically significant. This finding suggests that by this time, enough dimerized and internalized PD-L1 molecules have accumulated intracellularly to be detected by both flow cytometry (Figure 5) and confocal microscopy (Figure A1). A similar pattern was observed after 60 min of treatment. One possible explanation is that recycled PD-L1 molecules return to the cell surface and subsequently lose the surface-bound primary antibody during the acid-wash step. These observations are consistent with the confocal microscopy data. To test this hypothesis, additional experiments are underway using inhibitors of lysosomal/endosomal trafficking and recycling pathways to determine whether the observed phenomenon is driven by intracellular recycling and to further elucidate the underlying mechanism.

## 4. Discussion

The primary mechanism of BMS-202 involves the induction of PD-L1 dimerization [9,18,19,20,21]. Structural studies have shown that BMS-202 binds at the interface of two PD-L1 monomers, stabilizing a homodimeric state that sterically hinders interaction with the PD-1 receptor [18]. While the biophysical aspects of this dimerization are well-documented, its implications for cellular trafficking remain less explored. For PD-L1 dimerizers, reports are conflicting; some suggest rapid endocytosis and degradation [19,20], while others report stable surface levels after 24 h [9]. Our results suggest that internalization is extremely rapid and transient. Consequently, the 5 min peak may be missed in studies focusing on 4 h or 24 h time points.

PD-L1 is known to undergo constitutive endocytosis and recycling, a process heavily regulated by the transmembrane protein CMTM6 [17]. CMTM6 colocalizes with PD-L1 in recycling endosomes and protects it from being diverted to the lysosome for degradation, thereby maintaining high surface expression [17].

PD-L1 turnover is managed through two main pathways: the lysosomal and the proteasomal. Lysosomal degradation often follows the transition from early endosomes to multivesicular bodies and late endosomes, a process that can be accelerated by certain inhibitors such as S4-1 or targeted molecular constructs like LTzC [19,20]. Conversely, the proteasomal pathway involves the ubiquitination of the PD-L1 cytoplasmic tail by E3 ligases, a process inhibited by CMTM6 [17]. The fact that we did not observe a sustained decrease in total PD-L1 levels suggests that the BMS-202-induced dimers in our model may bypass these degradative pathways.

Notably, data for BMS-202 across cell lines demonstrate significant variations in surface PD-L1 dynamics [17,20]. While our time-course data suggests internalization, definitive proof awaits confirmation via imaging-based techniques. These discrepancies indicate that BMS-202 may operate via different modes of action depending on the duration of exposure and the affected cell line [18,20]. Our study demonstrates a new temporal effect of BMS-202 on RKO colon cancer cells, adding to the complex landscape of PD-L1 physiology. This highlights the importance of testing immediate-early events following exposure to BMS-202, as these initial events can dictate downstream cellular responses. Therefore, understanding the kinetics of surface PD-L1 recovery is critical for developing next-generation small-molecule inhibitors, as their efficacy may depend not only on blocking the PD-1 interface but also on overcoming the recycling machinery.

## 5. Conclusions

In this study, treatment of RKO cells with a non-cytotoxic concentration of BMS-202 (5 µM) produced a transient reduction in surface PD-L1, with the lowest levels observed at 15 min and a return to baseline by 24 h. Using a low-pH wash internalization assay, we observed a modest, non-statistically significant increase in intracellular PD-L1 signal at 15 and 60 min, consistent with, but not conclusive proof of, internalization of dimerized PD-L1 during this window. The percentage of PD-L1-positive cells remained relatively stable across the time course even as MFI declined, suggesting that the change in surface signal is better described as a reduction in receptor density per cell than as loss of PD-L1 positivity across the population. We did not observe a sustained decrease in total PD-L1 levels, which may indicate that the internalized fraction is recycled back to the surface rather than degraded, though this was not directly tested and remains a hypothesis to be examined with recycling and degradation pathway inhibitors. Overall, these results indicate that BMS-202 exposure is associated with an early, reversible change in surface PD-L1 levels on RKO cells within the first 60 min of treatment. Confirmation of the underlying trafficking mechanism, and assessment of whether this pattern generalizes to other PD-L1-expressing cell lines and other PD-L1 dimerizers, will require further experimental work.

## Data Availability

The original contributions presented in this study are included in the article. Further inquiries can be directed to the corresponding author.

## Abbreviations

The following abbreviations are used in this manuscript:

PD-L1	Programmed death-ligand 1
PD-1	Programmed death-1
EMT	Epithelial–mesenchymal transition
FDA	Food and Drug Administration
PI	Propidium Iodide
mAb	monoclonal antibody
IFN-γ	interferon-gamma
MFI	mean fluorescence intensity
